# Efficacy and safety of neoadjuvant chemotherapy regimens for triple-negative breast cancer: a network meta-analysis

**DOI:** 10.18632/aging.102188

**Published:** 2019-08-24

**Authors:** Yunhai Li, Dejuan Yang, Ping Chen, Xuedong Yin, Jiazheng Sun, Hongzhong Li, Guosheng Ren

**Affiliations:** 1 Department of Endocrine and Breast Surgery, The First Affiliated Hospital of Chongqing Medical University, Chongqing, China; 2 Chongqing Key Laboratory of Molecular Oncology and Epigenetics, The First Affiliated Hospital of Chongqing Medical University, Chongqing, China; 3 Department of Rheumatology, Daping Hospital, The Third Affiliated Hospital of Third Military Medical University, Chongqing, China

**Keywords:** network meta-analysis, triple-negative breast cancer, neoadjuvant chemotherapy, pathological complete response

## Abstract

Different neoadjuvant chemotherapies are available for triple-negative breast cancer (TNBC). Here, we performed a network meta-analysis to evaluate the pathological complete response (pCR) benefit and safety of treatment regimens. Pairwise and Bayesian network meta-analyses were performed to compare direct and indirect evidence, respectively. Twenty-three studies involving 12 regimens namely standard chemotherapeutic agents, bevacizumab (B)-, platinum salts (P)-, B plus P (BP)-, poly(ADP-ribose) polymerase inhibitors (Pi)-, P plus Pi (PPi)-, capecitabine (Ca)-, gemcitabine (Ge)-, zoledronic acid (Za)-, everolimus (E)-, P plus E (PE)-, and gefitinib (G)-containing regimens. The results showed that P-, B-, PPi-, and Za-containing regimens achieved higher pCR than standard chemotherapeutic agents. BP-containing regimens had a better pCR than B-containing regimens. In indirect comparisons, Za-, BP-, P-, and B-containing regimens were the top four strategies with the highest probability for pCR. Benefit-risk analysis showed that B-containing regimens had the highest acceptability of being the best treatment for better pCR achievement with fewer SAEs. The addition of P, B, BP, PPi, and Za to standard chemotherapeutic agents enhanced the pCR, but a balance between efficacy and safety should be carefully considered. B-containing regimens might be the best choice for neoadjuvant chemotherapy due to its better efficacy and tolerability.

## INTRODUCTION

Triple-negative breast cancer (TNBC) accounts for ~12%–17% of all breast cancers [1]. Because endocrine and anti-HER2 therapies are not suitable for TNBC patients, adjuvant chemotherapy is generally the only line of systemic treatment [1, 2]. Compared to other subtypes of breast cancer (e.g., hormone receptor or HER2 positive), TNBC patients suffer worse clinical outcomes [3]. In patients with early stage TNBC, neoadjuvant chemotherapy has become a standard approach and is more likely to achieve pathological complete response (pCR) than non-TNBC patients [4]. Event-free survival and overall survival is dramatically improved in TNBC patients who achieve a pCR after neoadjuvant chemotherapy, whereas patients with residual invasive disease after treatment have a high risk of recurrence [5].

Although there is a significant association between pCR and survival outcomes, the overall prognosis of TNBC remains unsatisfactory, and only ~30% of TNBC patients achieve pCR following treatment with standard anthracycline-, cyclophosphamide-, taxane-, and/or fluorouracil-based neoadjuvant chemotherapy [6]. Currently, different strategies are applied to increase TNBC pCR rates. For instance, adding platinum salts, which induce double-stranded DNA breaks and subsequent cell death, to standard neoadjuvant chemotherapy has been explored by several randomized control trials (RCTs), and has shown a promising pCR benefit in TNBC patients, especially in those with BRCA mutations [7–9]. However, severe toxicities resulting in hematological, gastrointestinal, and nervous system disorders have frequently been observed in patients treated with platinum-containing regimens [9]. Bevacizumab, a monoclonal antibody targeting vascular endothelial growth factor A, has been demonstrated to improve pCR rates when added to neoadjuvant chemotherapy in TNBC [10]. In I-SPY 2, a multicenter phase 2 trial, the addition of veliparib and carboplatin to standard neoadjuvant chemotherapy significantly increased the pCR proportion in TNBC patients compared with controls [11]. However, the NeoPARP study found no difference in pCR between an iniparib plus paclitaxel regimen vs. paclitaxel alone in TNBC [12].

Despite these results, the optimal regimen for TNBC treatment remains controversial due to the limitations of RCTs and conventional meta-analyses in comparing and integrating the efficacy of all available regimens. Network meta-analysis, also known as multiple-treatments or mixed-treatment analysis, not only allows the integration of the evidence without head-to-head comparison, but also can determine the superiority of different interventions by ranking probability and acceptability [13]. Therefore, we conducted a Bayesian network meta-analysis to estimate the efficacy and safety of the currently available neoadjuvant chemotherapies in TNBC. Here, we provide a systematic summary of different regimens that may aid in treatment decisions and future studies.

## RESULTS

### Overview of literature search and study characteristics

**Figure 1:**
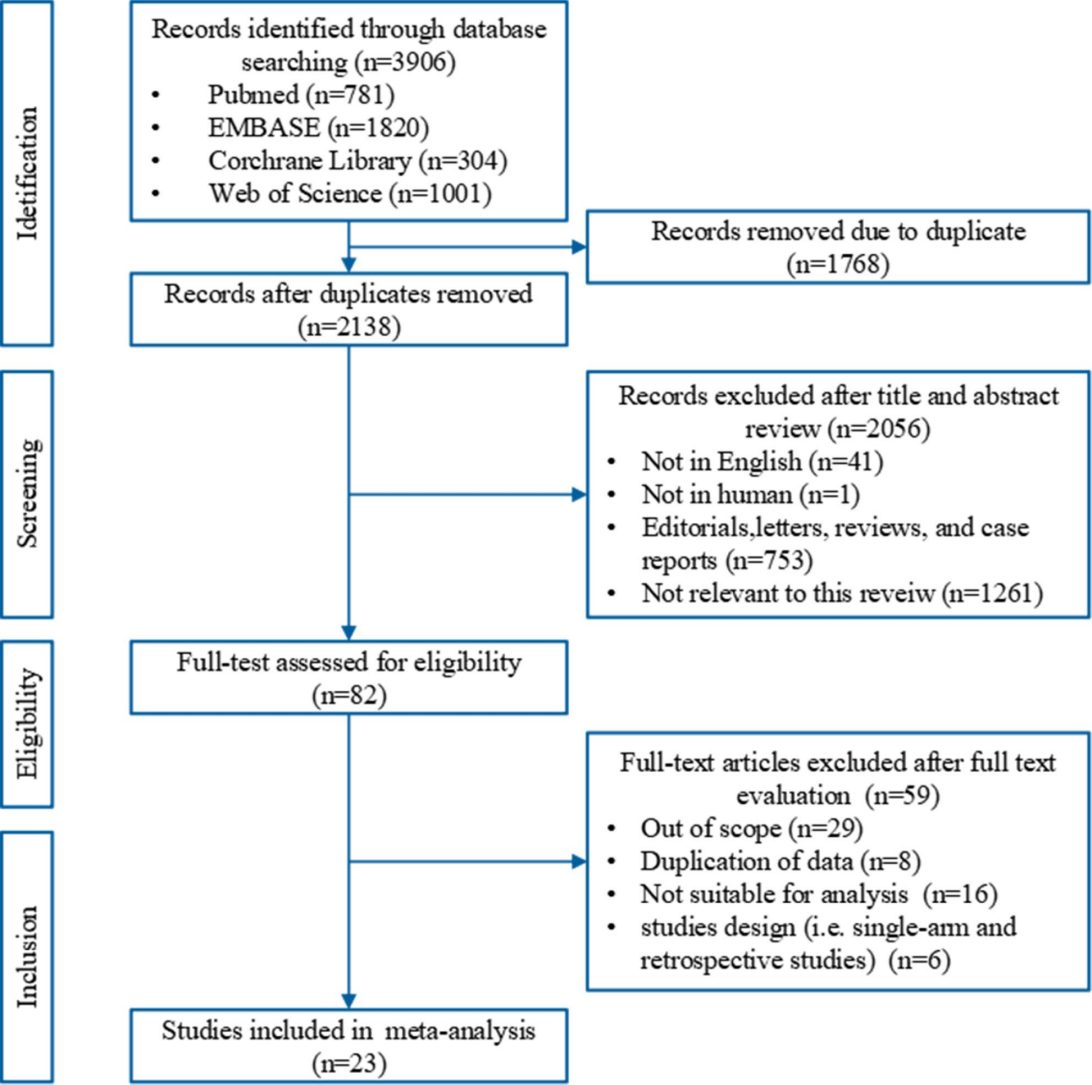
**A PRISMA flow chart of the literature search and study selection in this meta-analysis.**

A total of 2,138 relevant records were identified in the electronic databases. Of these, 82 potentially eligible studies were reviewed with full text. Fifty-nine studies were excluded because 29 were out of scope, eight were duplicated data, 16 were not suitable for analysis, four were retrospective studies, and two were single-armed studies. However, although the study by Enriquez et al. [14] is a single-armed trial, it was controlled using historical patient data; this study was therefore included in our analysis. All together, 23 studies [7–12, 15–30] comprising 4,099 patients were included. Twenty-one studies were reported as full text publications and two [14, 25] as conference abstracts. The PRISMA flow chart of study selection is shown in Figure 1.

**Figure 2:**
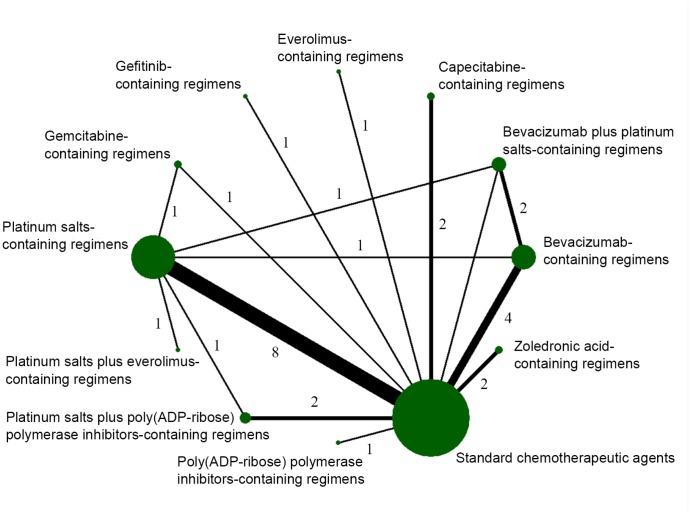
**Network diagram of eligible comparisons included in the network meta-analysis for pathological complete response (pCR).** The node size is proportional to the total number of patients in the regimen. The width of each line is proportional to the number of studies comparing the two regimens linked by the line.

All eligible studies were published between 2010 and 2018, and patients were enrolled between 2003 and 2017. Except for the historical controlled trial (HCT) [14], all studies were randomized controlled trials (RCTs). There were six phase 3 and 17 phase 2 trials. The pCR was defined as ypT0/is ypN0 in 20 studies, whereas in three trials [15, 17, 24] pCR was defined as ypT0/is. The main characteristics of the included studies are summarized in Table 1. Twelve treatment regimens were assessed: standard chemotherapeutic agents, bevacizumab (B)-, platinum salts (P)-, B plus P (BP)-, poly(ADP-ribose) polymerase inhibitors (Pi)-, P plus Pi (PPi)-, capecitabine (Ca)-, gemcitabine (Ge)-, zoledronic acid (Za)-, everolimus (E)-, P plus E (PE)-, and gefitinib (G)-containing regimens (Figure 2). As for the four-armed GeparTrio trial [16], we only included the patients in the two arms without early response, as the other two arms compared the efficacy between four and six cycles of standard chemotherapeutic agents. Moreover, vinorelbine in the Ge-containing arm was ignored in the GeparTrio trial. In the SOLTI NeoPARP [12] trial, we combined the two arms that used different dosages of iniparib into the Pi-containing regimen for the final analysis. The treatment details of the included trials are shown in Supplementary Table 1.

**Table 1:** Characteristics of eligible studies.

**Study**	**First author country**	**Type of trail**	**Trial phase**	**Masking**	**Recruitment period**	**No. of center**	**Arms**	**TNBC definition**	**Clinical stage**	**No. of patients analyzed**	**Trial name/registry number**
Aft 2010	America	Prospective RCT	II	Open-label	2003–2006	Single	2	ER/PR=0%, HER2= –; 1+; 2+/Hish–	II-III	40	NCT00242203
Houber 2010	Switzerland	Prospective RCT	III	Open-label	2002–2005	Multiple	4	ER/PR<10%, HER2= –; 1+; 2+/Hish–	II-III	89	GeparTrio/NCT00544765
Bernsdorf 2011	Sweden	Prospective RCT	II	Double-blind	2004–2007	Multiple	2	NA	II-III	82	NCT 00239343
Alba 2012	Spain	Prospective RCT	II	Open-label	2007–2010	Multiple	2	ER/PR≤1%, HER2= –; 1+; 2+/Hish-	II-III	93	GEICAM/2006-03/NCT00432172
Gerber 2013	Germany	Prospective RCT	III	Open-label	2007–2010	Multiple	2	ER/PR<10%, HER2= –; 1+; 2+/Hish–	II-III	663	GeparQuinto/GBG 44/NCT00567554
Ando 2014	Japan	Prospective RCT	II	Open-label	2010–2011	Multiple	2	ER/PR<10%, HER2= –; 1+; 2+/Hish–	II-III	75	NA
Earl 2014	UK	Prospective RCT	III	Open-label	2005–2007	Multiple	4	ER/PR-NA; HER2= –; 1+; 2+/Hish–	II-III	157	Neo-tAnGo/ NCT00070278
Gonzalez-Angulo 2014	America	Prospective RCT	II	Open-label	NA	Single	2	ER/PR≤5%; HER2= –; 1+; 2+/Hish-	II-III	50	NCT00499603
Steger 2014	Austria	Prospective RCT	III	Open-label	2004–2008	Multiple	2	ER/PR<10%, HER2= –; 1+; 2+/Hish–	Non-IV	127	ABCSG-24/NCT00309556
von Minckwitz 2014	Germany	Prospective RCT	II	Open-label	2011–2012	Multiple	2	ER/PR<1%, HER2= –; 1+; 2+/Hish–	II-III	315	GeparSixto-GBG 66/NCT01426880
Earl 2015	UK	Prospective RCT	III	Open-label	2009–2013	Multiple	2	ER/PR score=0–2/8; HER2= –; 1+; 2+/Hish–	II-III	241	ARTemis/NCT01093235
Hasegawa 2015	Japan	Prospective RCT	II	Open-label	2010–2012	Multiple	2	ER/PR-NA; HER2= –; 1+; 2+/Hish–	II-III	34	JONIE
Llombart-Cussac 2015	Spain	Prospective RCT	II	Open-label	2010–2011	Multiple	3	ER/PR<10%, HER2= –; 1+; 2+/Hish–	II-III	140	SOLTI NeoPARP/ NCT01204125
Martinez 2015	Mexico	Prospective RCT	II	Open-label	NA	NA	2	NA	NA	61	NA
Sikov 2015	America	Prospective RCT	II	Open-label	2009–2012	Multiple	4	ER/PR≤10%, HER2= –; 1+; 2+/Hish–	II-III	433	CALGB 40603
Nahleh 2016	America	Prospective RCT	II	Open-label	2010–2012	Multiple	2	ER/PR<1%, HER2= –; 1+; 2+/Hish–	II-III	67	SWOG S0800/ NCT00856492
Zhang 2016	China	Prospective RCT	II	Open-label	2006–2012	NA	2	ER/PR<10%, HER2= –; 1+; 2+/Hish–	II-III	87	NCT01276769
Rugo 2016	America	Prospective RCT	II	Open-label	2010–2012	Multiple	2	Special definition	II-III	60	I-SPY 2/NCT01042379
Enriquez 2017	Peru	Prospective HCT	II	Open-label	2013–2014	Single	2	NA	II-III	61	NA?
Gluz 2017	Germany	Prospective RCT	II	Open-label	2013–2015	Multiple	2	ER/PR<1%, HER2= –; 1+; 2+/Hish–	Non-IV	324	WSG-ADAPT TN/NCT01815242
Jovanović 2017	America	Prospective RCT	II	Double-blind	2009–2013	Multiple	2	ER/PR<10%, HER2= –; 1+; 2+/Hish–	II-III	145	NCT00242203
Loibl 2018	Germany	Prospective RCT	III	Double-blind	2014–2016	Multiple	3	ER/PR<1%, HER2= –; 1+; 2+/Hish–	II-III	634	BrighTNess/NCT02032277
Wu 2018	China	Prospective RCT	II	Open-label	2014–2017	Single	2	ER/PR<10%, HER2= –; 1+; 2+/Hish–	I-III	121	ChiCTR-TRC-14005019

RCT, randomized controlled trail; HCT, historical controlled trial; NA, not available.

The quality of evidence was evaluated using the Cochrane risk of bias tool [46]. There was low risk of bias for the majority of categories. However, caution should be taken for the HCT [14] due to its high risk of selection bias. The results of quality assessment are shown in Supplementary Table 2.

### Pairwise meta-analysis of primary outcome

**Figure 3:**
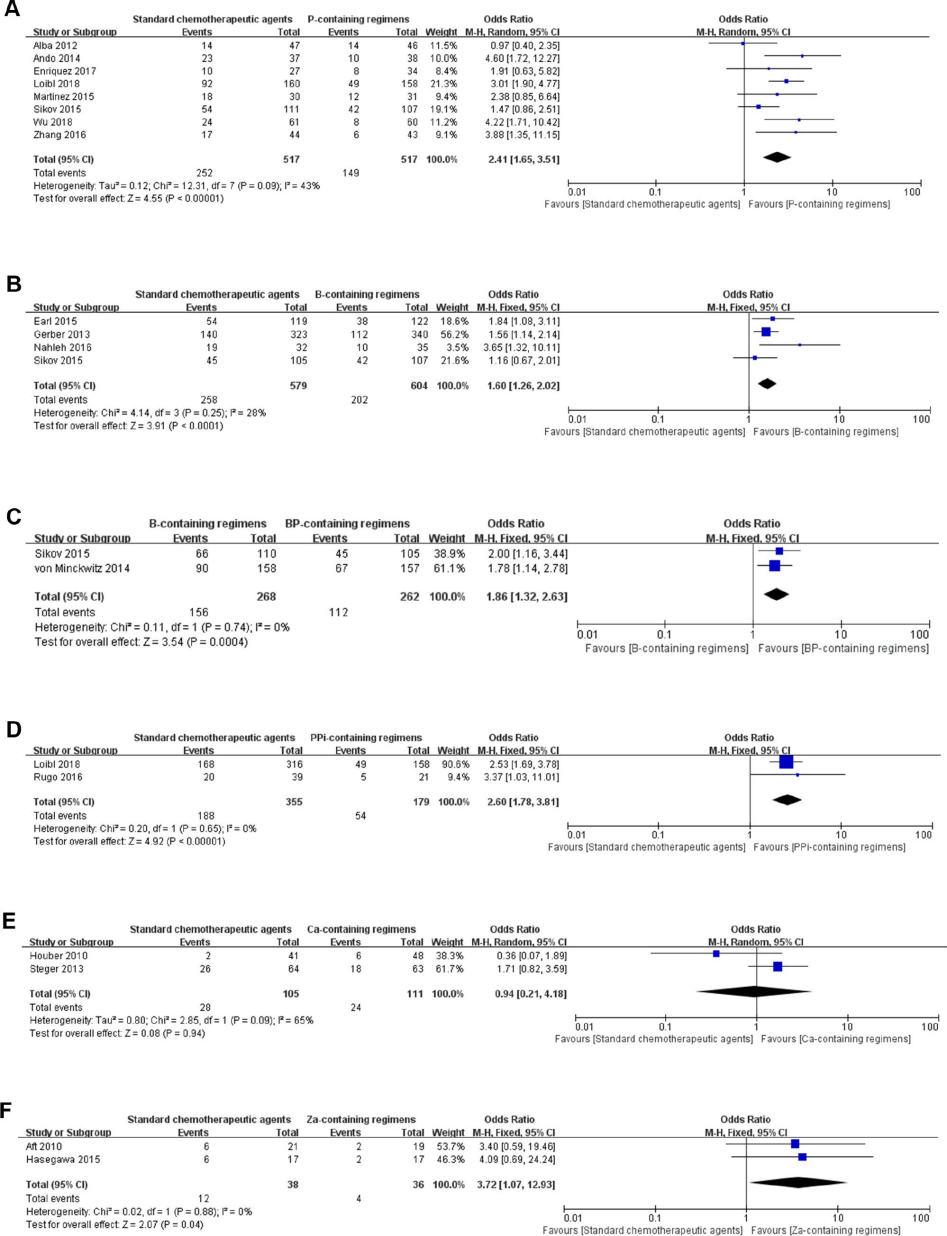
**Forest plots of pair-wise meta-analyses for pathological complete response (pCR).** (**A**) Standard chemotherapeutic agents vs. P-containing regimens. (**B**) Standard chemotherapeutic agents vs. B-containing regimens. (**C**) B-containing regimens vs. BP-containing regimens. (**D**) Standard chemotherapeutic agents vs. PPi-containing regimens. (**E**) Standard chemotherapeutic agents vs. Ca-containing regimens. (**F**) Standard chemotherapeutic agents vs. Za-containing regimens.

There were eight studies that directly compared standard chemotherapeutic agents with P-containing regimens. Because a modest heterogeneity (*p*=0.09, *I^2^*=43%) was detected, a pairwise comparison was performed with a random-effects model. We found that P-containing regimens were associated with a significant pCR benefit compared to standard chemotherapeutic agents (OR=2.41, 95% CI: 1.65–3.51, *p*<0.00001; Figure 3A). B-containing regimens had a better pCR than standard chemotherapeutic agents (OR=1.60, 95% CI: 1.26–2.02, *p*<0.0001) as analyzed by fixed-effect model (*p*=0.25, *I^2^*=28%; Figure 3B). BP-containing regimens were more effective than B-containing regimens (OR=1.86, 95% CI: 1.32–2.63, *p*=0.0004; Figure 3C). Two studies compared PPi-containing regimens with standard chemotherapeutic agents (OR=2.60, 95% CI: 1.78–3.81, *p*<0.00001), which indicated that PPi-containing regimens were superior in pCR achieving (Figure 3D); however, caution should be taken because the weights of the two studies were 90.6% and 9.4%, respectively. There was no significant difference between standard chemotherapeutic agents and Ca-containing regimens (OR=0.94, 95% CI: 0.21–4.18, *p*=0.94; Figure 3E). Za-containing regimens showed a significant pCR benefit (OR=3.72, 95% CI: 1.07–12.93, *p*=0.04) compared to standard chemotherapeutic agents (Figure 3F).

Sensitivity analyses were conducted to detect the influence of individual studies on the comparisons of standard chemotherapeutic agents with P- and B-containing regimens by omitting one study at a time. Omission of any single study did not materially alter the pooled effects (data not shown). The shapes of funnel plots of the two comparisons were close to symmetric, and no significant publication bias was identified by Begg's and Egger's tests (Supplementary Figure 1).

### Bayesian network meta-analysis of primary outcome

**Figure 4:**
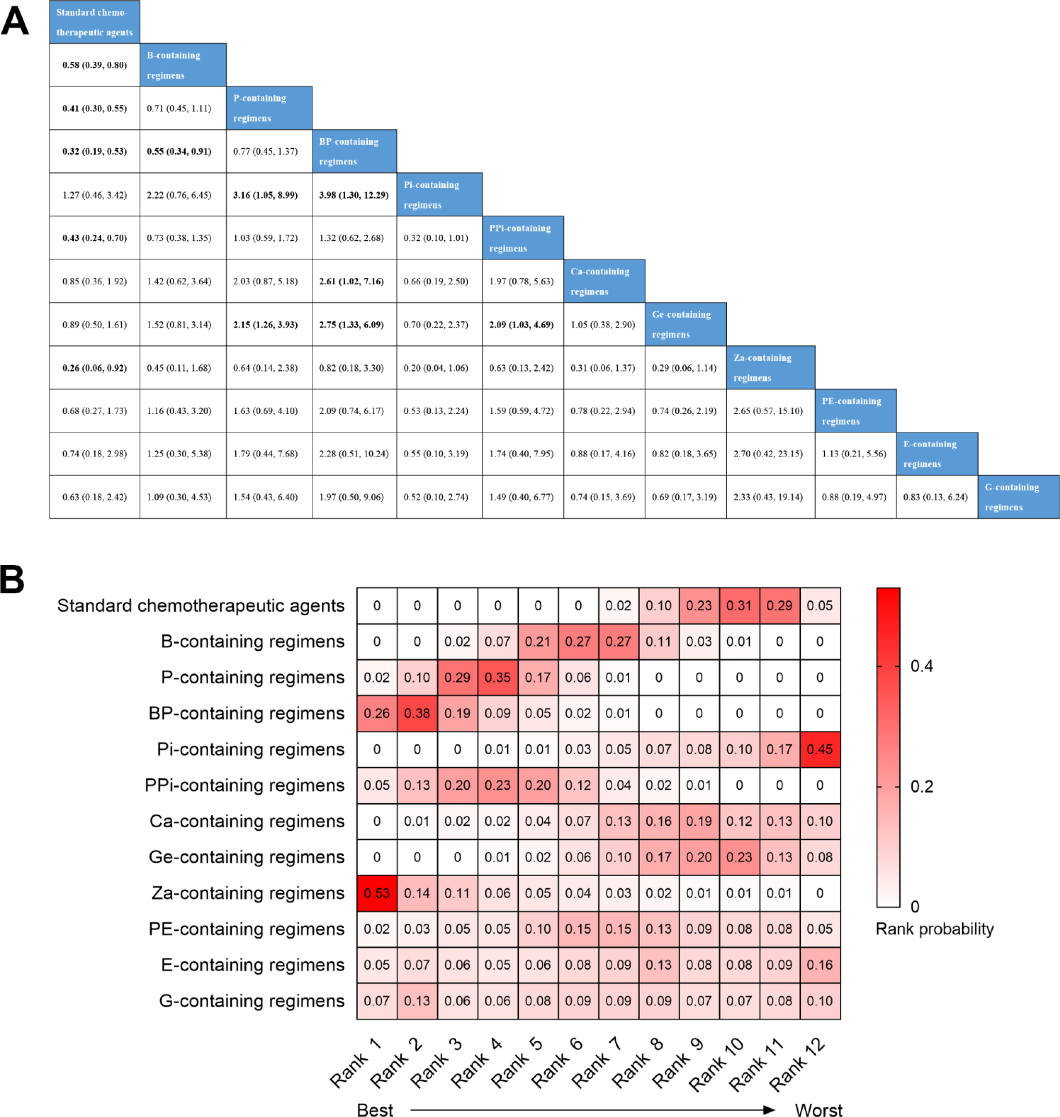
**Bayesian network meta-analysis for pathological complete response (pCR).** (**A**) The league table of comparisons. Data are presented as odds radio (OR) and 95% confidence intervals (CI). An OR>1 favors the column-defining treatment, and an OR<1 favors the row-defining treatment. (**B**) Heatmap of the rank probability of the twelve regimens for pCR. Rank 1 represents the best treatment and rank 12 represents the worst. Rank probabilities sum to one, both within a rank over treatments and within a treatment over ranks.

All of the 23 trials were included in the network meta-analysis for pCR. Node-splitting analysis was performed in order to evaluate the inconsistency, and no statistical difference was identified between direct and indirect evidence (data not shown). Therefore, the Bayesian network meta-analysis was conducted with a consistency model (Figure 4A). The results showed that the pCR incidence achieved by standard chemotherapeutic agents was significantly lower than B- (OR=0.58, 95% CI: 0.39–0.80), P- (OR=0.41, 95% CI: 0.30–0.55), BP- (OR=0.32, 95% CI: 0.19–0.53), PPi- (OR=0.43, 95% CI: 0.24–0.70), and Za-containing regimens (OR=0.26, 95% CI: 0.06–0.92). B-containing regimens had a poor pCR than BP-containing regimens (OR=0.55, 95% CI: 0.34–0.91). P-containing regimens were significantly related to pCR benefit compared with Pi- (OR=3.16, 95% CI: 1.05–8.99) and Ge-containing regimens (OR=2.15, 95% CI: 1.26–3.93). BP-containing regimens showed a significantly higher rate pCR than Pi- (OR=3.98, 95% CI: 1.30–12.29), Ca- (OR=2.61, 95% CI: 1.02–7.16), and Ge-containing regimens (OR=2.75, 95% CI: 1.33–6.09). Moreover, PPi-containing regimens showed a significant pCR advantage over Ge-containing regimens (OR=2.09, 95% CI: 1.03–4.69). Due to the pCR definition bias of three studies [15, 17, 24] and selection bias of the HCT [14], we further performed a subgroup analysis that excluded the regimens of the four studies. However, no significant change was observed for the positive results identified above (Supplementary Figure 2A), which demonstrated the robustness of these results.

The rank probability of regimens was also analyzed. As shown in Figure 4B, the three best treatments were Za-containing regimens (53%), BP-containing regimens (38%), and P-containing regimens (29%) ranked first to third in pCR achieving, while Pi-containing regimens (45%), standard chemotherapeutic agents (29%), and Ge-containing regimens (23%) proved to be the three worst treatments, occupying rank 12 to rank 10. In the subgroup analysis of regimens without Za-containing regimens, BP-containing regimens (64%) were the best treatment for pCR followed by P-containing regimens (41%) and PPi-containing regimens (30%). Consistently, Pi-containing regimens (47%), standard chemotherapeutic agents (27%), and Ge-containing regimens (20%) remained the three worst treatments (Supplementary Figure 2B).

### Meta-analysis for grade 3–4 hematological adverse events

There were 11 trails with eight regimens that reported anemia, 16 trials with 10 regimens that reported neutropenia, and 13 trials with seven regimens that reported thrombocytopenia (Supplementary Table 3 and Supplementary Figure 3). We found that P-containing regimens were associated with higher risk of anemia (OR=16.49, 95% CI: 7.52–36.14, *p*<0.00001), neutropenia (OR=3.30, 95% CI: 1.35–8.08, *p*=0.009), and thrombocytopenia (OR=12.93, 95% CI: 5.91–28.29, *p*<0.00001) compared with standard chemotherapeutic agents (Supplementary Figure 4A, 4B, and 4D). Consistently, subgroup analyses based on TNBC patients also revealed that P-containing regimens resulted in a higher incidence of anemia (OR=15.54, 95% CI: 6.64–36.34, *p*<0.00001), neutropenia (OR=3.36, 95% CI: 1.04–10.83, *p*=0.04) and thrombocytopenia (OR=13.84, 95% CI: 6.14–31.20, *p*<0.00001), compared to standard chemotherapeutic agents (Supplementary Figure 5A–5C). B-containing regimens were associated with a higher risk of neutropenia (OR=1.20, 95% CI: 1.01–1.43, *p*=0.04) than standard chemotherapeutic agents (Supplementary Figure 4C). No significant bias was identified by funnel plots or Begg's and Egger's tests (Supplementary Figure 6).

**Figure 5:**
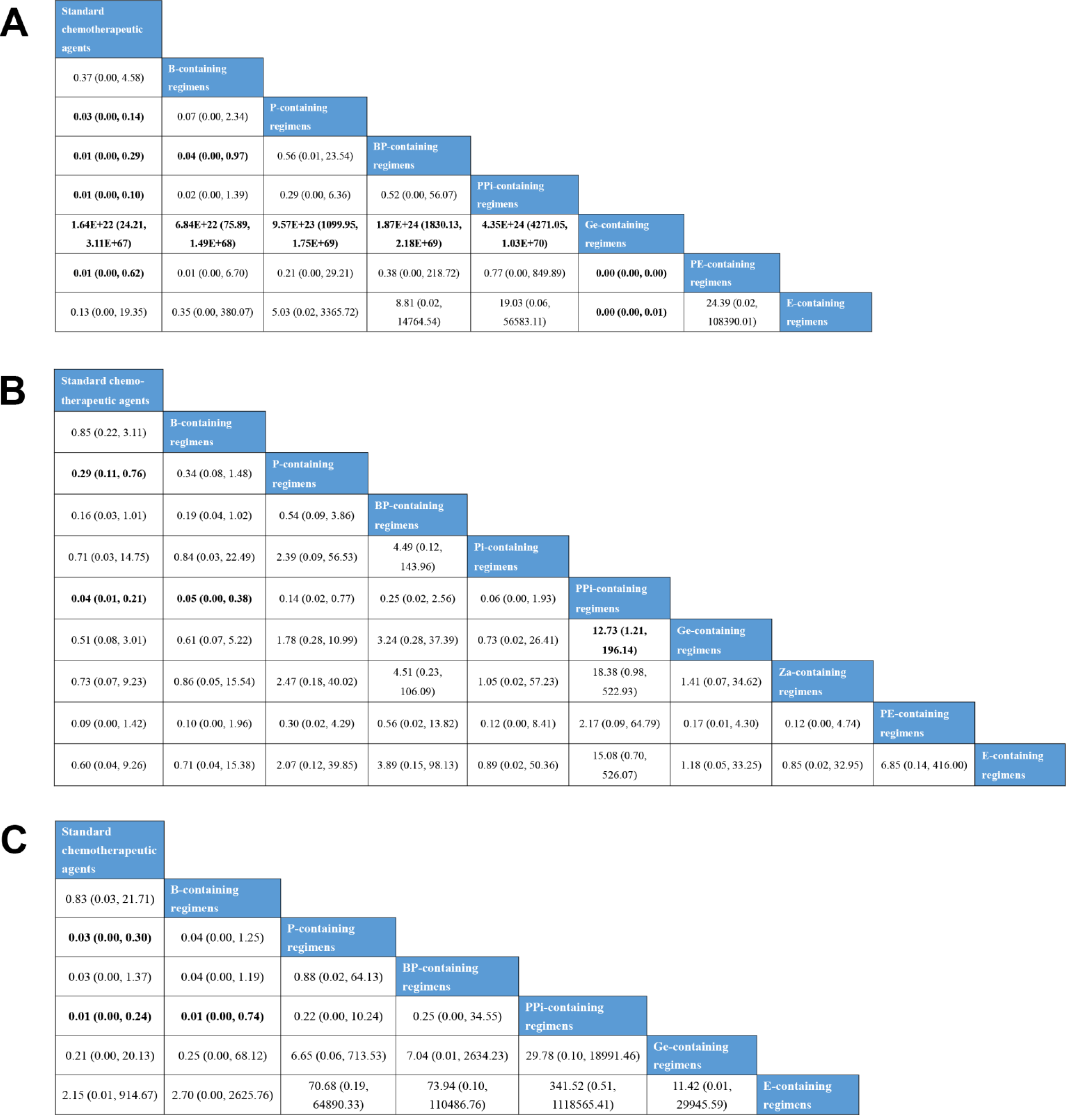
**Bayesian network meta-analysis for grade 3–4 hematological adverse events.** (**A**) The league table for comparisons of anemia. (**B**) The league table for comparisons of neutropenia. (**C**) The league table for comparisons of thrombocytopenia. Data are presented as odds radio (OR) and 95% confidence intervals (CI). An OR>1 favors the row-defining treatment, and OR<1 favors the column-defining treatment.

Indirect comparisons were then performed using a consistency model, as node-splitting analyses revealed no significant difference between the direct and indirect evidence for anemia, neutropenia, and thrombocytopenia (Figure 5). With respect to anemia, standard chemotherapeutic agents (OR=1.64E+22, 95% CI: 24.21–3.11E+67), B- (OR=6.84E+22, 95% CI: 75.89–1.49E+68), P- (OR=9.57E+23, 95% CI: 1099.95–1.75E+69), BP- (OR=1.87E+24, 95% CI: 1830.13–2.18E+69), PPi- (OR=4.35E+24, 95% CI: 4271.05–1.03E+70), PE- (OR=0.00, 95% CI: 0.00–0.00), and E-containing regimens (OR=0.00, 95% CI: 0.00–0.01) were associated with higher incidences than Ge-containing regimens. P- (OR=0.03, 95% CI: 0.00–0.14), BP- (OR=0.01, 95% CI: 0.00–0.29), PPi- (OR=0.01, 95% CI: 0.00–0.10), and PE-containing regimens (OR=0.01, 95% CI: 0.00–0.62) induced more anemia events than standard chemotherapeutic agents. BP-containing regimens (OR=0.04, 95% CI: 0.00–0.97) induced more anemia events than B-containing regimens. Neutropenia was significantly lower in standard chemotherapeutic agents than in P- (OR=0.29, 95% CI: 0.11–0.76) and PPi-containing regimens (OR=0.04, 95% CI: 0.01–0.21). B- (OR=0.05, 95% CI: 0.00–0.38) and Ge-containing regimens (OR=12.73, 95% CI: 1.21–196.14) were associated with lower incidences of neutropenia compared to PPi-containing regimens. With respect to thrombocytopenia, P- (OR=0.03, 95% CI: 0.00–0.30) and PPi-containing regimens (OR=0.01, 95% CI: 0.00–0.24) were associated with a higher risk of incidence than standard chemotherapeutic agents. PPi-containing regimens also had a higher risk of thrombocytopenia compared to B-containing regimens (OR=0.01, 95% CI: 0.00–0.74).

The rank probability for each treatment inducing SAEs showed that Ge-containing regimens (99%) were the best treatment, resulting in the lowest incidence of anemia adverse events, whereas PE-containing regimens (45%) were found to be the worst treatment (Supplementary Figure 7A). PPi-containing regimens ranked first for the highest prevalence of neutropenia (60%) and thrombocytopenia (64%) events (Supplementary Figure 7B and 7C). Pi- (26%) and E-containing regimens (52%) had the lowest probability of leading to neutropenia and thrombocytopenia, respectively.

### Stochastic multi-criteria acceptability analysis (SMAA)

**Figure 6:**
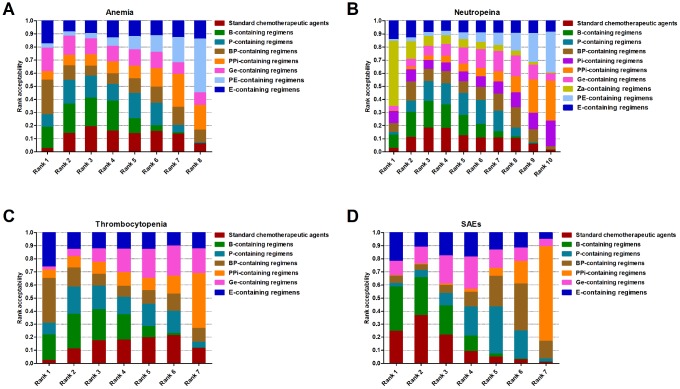
**Stochastic multi-criteria acceptability analysis for benefit-risk.** (**A**) Rank probability of regimens based on synthesizing pCR and anemia. (**B**) Rank probability of regimens based on synthesizing pCR and neutropenia. (**C**) Rank probability of regimens based on synthesizing pCR and thrombocytopenia. (**D**) Rank probability of regimens based on synthesizing pCR and the three serious adverse events. Rank 1 represents the best treatment and rank *N* represents the worst. The proportion corresponds to the probability of each regimen to be at a specific rank.

Benefit-risk analyses were performed by SMAA with missing preferences. Standard chemotherapeutic agents were set as the baseline. The analyses between pCR and anemia showed that BP-containing regimens had the highest acceptability (26.46%), ranking first (Figure 6A) with a CF=0.34 (Supplementary Figure 8A). Za-containing regimens (49.72%, Figure 6B) were the best treatment when considering pCR and neutropenia with a CF=0.62 (Supplementary Figure 8B). BP-containing regimens ranked first in acceptability (34.18%, CF=0.42), resulting in a higher pCR and fewer thrombocytopenia events (Figure 6C and Supplementary Figure 8C). When considering pCR with the three SAEs, B-containing regimens were the best choice with highest rank acceptability (34.01%, Figure 6D) and a CF=0.43 (Supplementary Figure 8D).

## DISCUSSION

A growing number of clinical trials are being performed in order to improve the effectiveness of neoadjuvant chemotherapies in TNBC by adding different drugs to the standard chemotherapeutic agents. However, results are controversial and remain isolated in the absence of systematic integration. Therefore, a comprehensive study was warranted to give a summary of the results from these publications. To the best of our knowledge, this is the first network meta-analysis to investigate the pCR efficacy and safety of neoadjuvant chemotherapy regimens in TNBC.

In the present study, we enrolled 23 clinical trials with 4,099 TNBC individuals assigned to 12 neoadjuvant chemotherapy regimens, aiming to identify which treatment was optimal in achieving higher pCR rates and resulting in fewer SAEs. The results of pairwise meta-analyses showed that the most highly studied P-containing regimens were significantly associated with better pCR rates, but worse SAEs, compared with standard chemotherapeutic agents. Consistently, two previous meta-analyses also revealed that platinum-based neoadjuvant chemotherapies clearly increased pCR rates compared with platinum-free neoadjuvant chemotherapies [31, 32]. Although no survival benefit was observed for platinum-based neoadjuvant chemotherapy as pooled by two RCTs [31], many more studies with long-term follow-up are required to clarify the potential association between survival outcomes and platinum salts. TNBC was demonstrated to be more sensitive to platinum salts than non-TNBC [32], with the probable reason being that TNBC is more commonly related to BRCA mutations or homologous recombination DNA repair deficiencies [33, 34]. PARP inhibitors can block DNA repair pathways, which are crucial for tumor cell survival in patients with BRCA mutations or homologous recombination DNA repair deficiencies [34]. Therefore, it is reasonable to speculate that PARP inhibitors might enhance the anti-tumor activity of cytotoxic agents resulting in DNA damage, such as platinum salts. However, in this study, although PPi-containing regimens significantly increased pCR rates compared to standard chemotherapeutic agents, there was no difference in efficacy between P- and PPi-containing regimens, indicating that PARP inhibitors did not enhance the effects of platinum salts. This result is consistent with the findings of BrighTNess trial [9]. Moreover, a benefit-risk analysis showed that PPi-containing regimens might be the worst treatment choice when considering pCR and SAEs. In addition, we found that Pi-containing regimens without platinum salts were not superior to any other regimen. Thus, our results do not support further investigation into the use of PARP inhibitors added to standard chemotherapeutic agents or in combination with platinum salts at the present dosage in TNBC patients.

Bevacizumab is another frequently studied agent in neoadjuvant chemotherapy for TNBC. It has shown clinical efficacy in prolonging progression-free-survival, but not overall survival, in metastatic TNBC [35]. In our work, we found that B-containing regimens were significantly associated with a higher pCR rate than standard chemotherapeutic agents, while only a modest correlation between B-containing regimens and neutropenia prevalence was detected. However, bevacizumab may lead to other adverse events in the circulatory, nervous or urinary systems [26, 35]. Consistent with our study, a recent network meta-analysis reported that bevacizumab plus chemotherapy significantly improved pCR of TNBC patients when compared with chemotherapy plus placebo [36]. Moreover, bevacizumab plus chemotherapy was demonstrated to be significantly associated with longer progression-free survival than chemotherapy alone in advanced/metastatic TNBC [37]. Although no significant different was detected between B- and P-containing regimens in pCR rates, the combination of bevacizumab and platinum salts (BP-containing regimens) was able to increase the efficacy of both B- and P-containing regimens, when compared with standard chemotherapeutic agents. Importantly, our benefit-risk analysis revealed that B-containing regimens might be the best treatment to achieve a relatively high pCR rate with fewer hematological SAEs.

To our surprise, zoledronic acid, a nitrogen-containing bisphosphonate that induces osteoclast apoptosis and inhibits bone resorption [38], showed a significant pCR benefit when added to standard chemotherapeutic agents. No statistical difference in SAEs incidence was observed between Za-containing regimens and standard chemotherapeutic agents. Moreover, Za-containing regimens had the highest probability of being the best treatment for achieving pCR. However, caution should be taken due to the limited number of subjects in the two original RCTs investigating Za-containing regimens, with only 40 and 34 patients, respectively [15, 24]. Although the effects of zoledronic acid on pCR rate were not striking in the two RCTs, the pooled effect was statistically significant, mainly due to the larger sample size in our work. Accordingly, we hold the opinion that further RCTs with larger sample sizes are needed to confirm any potential pCR benefit of zoledronic acid, and to strengthen our results. In addition to its anti-metastatic properties within bone tissue, the anti-tumor activities of zoledronic acid may be explained by several mechanisms, including inducing tumor cell apoptosis, enhancing the cytotoxic effects of chemotherapeutic agents, suppressing neoangiogenesis, and involving immunomodulation [39–42]. Moreover, disease-free survival benefits of zoledronic acid have also been detected in premenopausal endocrine-positive patients [43, 44]. However, the survival benefit of zoledronic acid in TNBC remains unclear.

There are several limitations of the present study. First, the sample sizes of several trials were relatively small. Specifically, the three RCTs involving Za-containing regimens and the study of E-containing regimens only included approximately 50 TNBC patients, which might weaken the effects or lead to false positive results. In addition, there were only one study for E-, PE, and Ge-containing regimens and the number of SAEs very relatively small in these studies, which might result in the wide 95% CIs; therefore, the results of SEAs regarding these regimens should be interpreted with caution. Second, there are several potential heterogeneities in the included studies, such as pCR definition, study design, and the specific components of standard chemotherapeutic agents. However, subgroup and sensitivity analyses based on pCR definition and study design demonstrated that the heterogeneities were unlikely to refute the overall results. Moreover, we integrated taxanes, anthracyclines, cyclophosphamide, and fluorouracil as comparative standard chemotherapeutic agents, as most control arms of the included studies were based on these four kinds of drugs, but differences still existed between studies. Therefore, consideration of these heterogeneities should be taken into account in the interpretation of our findings. Third, the kinds of SAEs reported in each study were different, and SAE data were not available for several trials. Thus, it was difficult to conduct a comprehensive analysis for all SAEs and regimens. Although there was no evidence showing a significant correlation between SAEs and the molecular subtype of breast cancer, there may be bias in the pooled effects of SAEs compared to the real results. Fourth, the correlation between neoadjuvant chemotherapy and survival outcomes was not evaluated due to the lack of survival data in most studies. However, we noticed that some RCTs are still ongoing and a secondary analysis focusing on long-term survival benefits may be reported in the near future. Therefore, a subsequent updated network meta-analysis will help to integrate the effects of these regimens on clinical outcomes.

Despite the aforementioned limitations, our study has for the first time systematically compared the pCR efficacy and hematological SAEs of the currently available neoadjuvant chemotherapy regimens in TNBC. In conclusion, this study demonstrated that Za-, BP-, P-, and B-containing regimens are the top four treatment strategies, showing a high efficacy in achieving pCR. When considering both efficacy and SAEs, B-containing regimens had the highest acceptability to be the best treatment for achieving a relatively high pCR with fewer SAEs. However, additional well-designed RCTs with larger sample sizes are required to strengthen the findings of this meta-analysis and further determine the survival benefits of these neoadjuvant therapies in TNBC.

## METHODS

### Search strategy

This meta-analysis was performed in accordance with the guideline of Preferred Reporting Items for Systematic Reviews and Meta-analyses (PRISMA) [45]. Literatures, published before May 31, 2018, were identified in PubMed, Embase, Cochrane library, and Web of Science. The following key words were used: (breast OR mammary) AND (cancer OR cancers OR tumor OR neoplasm OR carcinoma) AND (neoadjuvant chemotherapy OR induction chemotherapy OR pre-operative chemotherapy) AND (TNBC OR triple-negative OR triple negative OR basal-like OR HER2 negative) AND (pathological complete response OR pCR), without any restrictions. The reference lists from relevant studies, reviews, and meta-analyses were manually screened for potentially eligible publications.

### Selection criteria

The inclusion and exclusion criteria were prespecified. Eligible trials were studies prospectively comparing at least two arms of different neoadjuvant chemotherapy regimens in TNBC patients. Studies were excluded if they were: (1) non-human studies, letters, reviews, editorial comments or case reports; (2) single-arm or dosage-finding studies; (3) articles without raw data or with a sample size of less than 30; and (4) ongoing trials without reported results. If several publications from the same trial were identified, only the latest or complete publication was included. Two reviewers independently evaluated the risk of bias for eligible studies using the Cochrane Collaboration risk of bias tool [46]. Any discrepancies were resolved by discussing with all investigators.

### Data extraction

Data were extracted by two independent authors. The following information was recorded for eligible studies: first author name, publication year, country, study design, trial phase, recruitment period, masking, number of centers, TNBC definition, clinical stage of patients, and trial name or registry number. Treatment regimens, sample size, primary outcome (pCR), and secondary outcome (grade 3–4 hematological adverse events), if available, were also recorded.

### Treatment regimens and outcomes definition

Due to the widespread use of combinations of taxanes, anthracyclines, cyclophosphamide, and/or fluorouracil, we defined the regimen as standard chemotherapeutic agents, which consisted of treatment with certain or all four drugs. The addition of any other drugs based on the standard chemotherapeutic agents was regarded as a new regimen. In the case of multi-arm studies comparing different dosages of one agent with another, we combined the results of the same agent into one arm. Administration sequence and frequency were not considered in the present study. The primary outcome was pCR, which was defined as the absence of invasive breast cancer in the breast and axillary lymph nodes (ypT0/is ypN0). If ypT0/is ypN0 was not reported, ypT0/is, defined as the absence of invasive breast cancer in the breast, was substituted. Secondary outcomes were treatment-related serious (grade 3–4) adverse events (SAEs) in hematology, including anemia, neutropenia, and thrombocytopenia. As for SAEs, we hypothesized that there was no significant correlation between molecular subtypes and hematological adverse events. Therefore, if the SAEs of TNBC were not available, we included the number of SAEs from all molecular subtypes of breast cancer patients. If only the percentages of primary and secondary outcomes were reported, we calculated the number of events by multiplying percentages by the number of patients.

### Statistical methods

The odds ratio (OR) was utilized for estimating pooling effect sizes. For the pairwise meta-analysis, heterogeneity was calculated using Cochrane Q statistics and an *I*^2^ test. Statistical heterogeneity was defined as *p*<0.1 and/or *I^2^*>50%. A pairwise meta-analysis was conducted with a random-effects model or a fixed-effect model depending on the existence of statistical heterogeneity or not, respectively. All pairwise meta-analyses were performed by Review Manager software version 5.2. Results were reported as OR and 95% confidence intervals (CIs). All *p-*values were two-sided and a value less than 0.05 was considered statistically significant. Publication bias was detected by funnel plots, Begg’s and Egger’s tests using Stata software version 12.0.

A Bayesian network-meta analysis was performed by Aggregate Data Drug Information System (ADDIS) software, version 1.16.8 (http://www.drugis.org) [47]. The consistency between direct and indirect evidence was verified by node splitting analyses. If no significant inconsistency was detected, a consistency model was used to analyze the relative effects of the interventions. Otherwise, an inconsistency model was applied. Convergence was assessed using the Brooks-Gelman-Rubin method, which compared within-chain and between-chain variance to calculate the potential scale reduction factor (PSRF). We defined a PSRF of less than 1.05 as an indication of achieving convergence, and finished the simulation. Otherwise, the model should be extended until the PSRF is less than 1.05. The results of the network meta-analyses were presented as OR and 95% CI. Rank probability for each treatment was calculated to achieve the treatment ranking. Benefit-risk analyses were performed with synthesis evidence by stochastic multi-criteria acceptability analysis (SMAA) to jointly analyze the efficacy and SAEs for interventions. Rank acceptability and confidence factor (CF) were calculated with missing preference.

## Supplementary Material

Supplementary Figures

Supplementary Table 1

Supplementary Tables 2 and 3
