# Supplementary material for: Efficacy and safety of neoadjuvant chemotherapy regimens for triple-negative breast cancer: a network meta-analysis
Source: Aging (Albany NY). 2019 Aug 24;11(16):6286–311. doi: 10.18632/aging.102188 (PMC6738404; doi:10.18632/aging.102188)
Supplement: Supplementary Figures [file aging-11-102188-s003.pdf]

SUPPLEMENTARY FIGURES

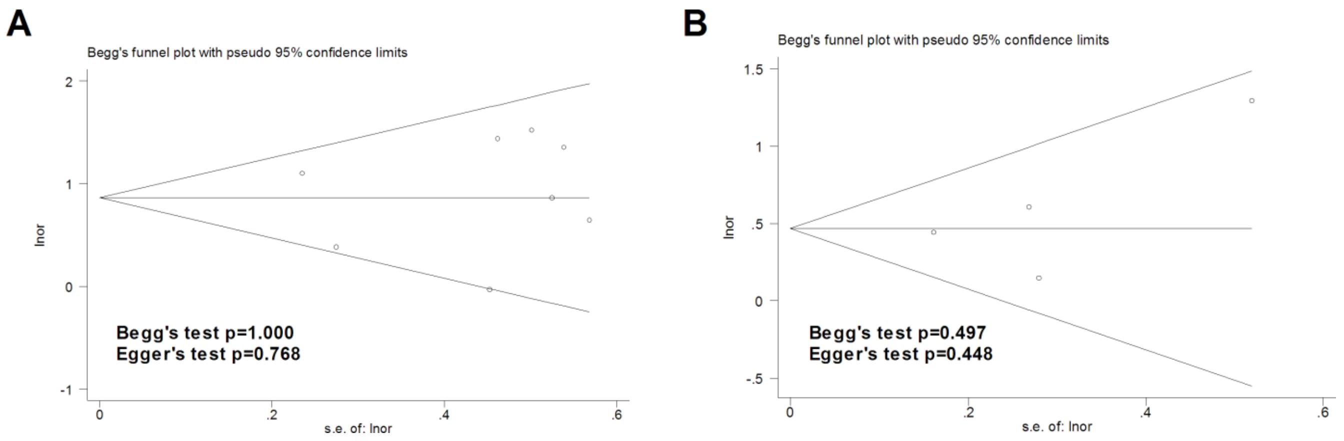

Supplementary Figure 1. Funnel plots of the publication bias tests for direct comparisons of pCR. (**A**) Standard chemotherapeutic agents vs. P-containing regimens. (**B**) Standard chemotherapeutic agents vs. B-containing regimens.

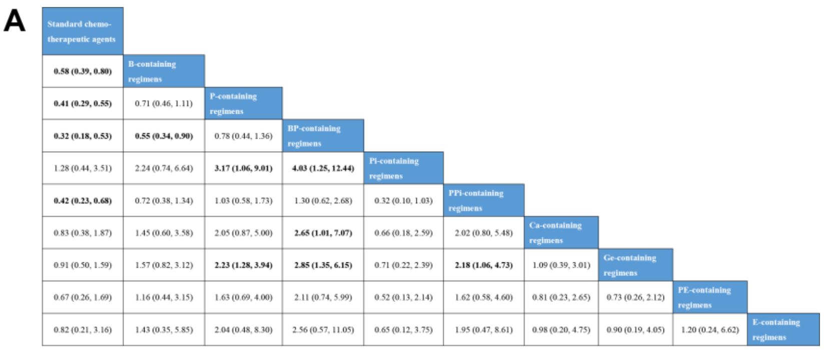
A

Standard chemo-therapeutic agents									
**0.58 (0.39, 0.80)**	B-containing regimens								
**0.41 (0.29, 0.55)**	0.71 (0.46, 1.11)	P-containing regimens							
**0.32 (0.18, 0.53)**	**0.55 (0.34, 0.90)**	0.78 (0.44, 1.36)	BP-containing regimens						
1.28 (0.44, 3.51)	2.24 (0.74, 6.64)	**3.17 (1.06, 9.01)**	**4.03 (1.25, 12.44)**	Pi-containing regimens					
**0.42 (0.23, 0.68)**	0.72 (0.38, 1.34)	1.03 (0.58, 1.73)	1.30 (0.62, 2.68)	0.32 (0.10, 1.03)	PPi-containing regimens				
0.83 (0.38, 1.87)	1.45 (0.60, 3.58)	2.05 (0.87, 5.00)	**2.65 (1.01, 7.07)**	0.66 (0.18, 2.59)	2.02 (0.80, 5.48)	Ca-containing regimens			
0.91 (0.50, 1.59)	1.57 (0.82, 3.12)	**2.23 (1.28, 3.94)**	**2.85 (1.35, 6.15)**	0.71 (0.22, 2.39)	**2.18 (1.06, 4.73)**	1.09 (0.39, 3.01)	Ge-containing regimens		
0.67 (0.26, 1.69)	1.16 (0.44, 3.15)	1.63 (0.69, 4.00)	2.11 (0.74, 5.99)	0.52 (0.13, 2.14)	1.62 (0.58, 4.60)	0.81 (0.23, 2.65)	0.73 (0.26, 2.12)	PE-containing regimens	
0.82 (0.21, 3.16)	1.43 (0.35, 5.85)	2.04 (0.48, 8.30)	2.56 (0.57, 11.05)	0.65 (0.12, 3.75)	1.95 (0.47, 8.61)	0.98 (0.20, 4.75)	0.90 (0.19, 4.05)	1.20 (0.24, 6.62)	E-containing regimens

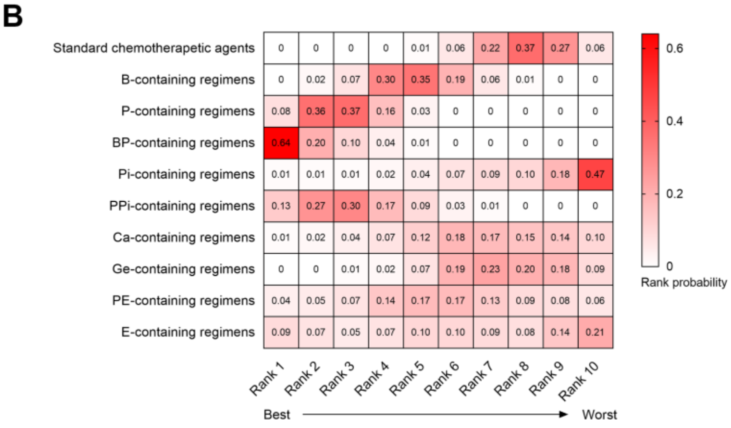

	Rank 1	Rank 2	Rank 3	Rank 4	Rank 5	Rank 6	Rank 7	Rank 8	Rank 9	Rank 10
Standard chemotherapetic agents	0	0	0	0	0.01	0.06	0.22	0.37	0.27	0.06
B-containing regimens	0	0.02	0.07	0.30	0.35	0.19	0.06	0.01	0	0
P-containing regimens	0.08	0.36	0.37	0.16	0.03	0	0	0	0	0
BP-containing regimens	0.64	0.20	0.10	0.04	0.01	0	0	0	0	0
Pi-containing regimens	0.01	0.01	0.01	0.02	0.04	0.07	0.09	0.10	0.18	0.47
PPi-containing regimens	0.13	0.27	0.30	0.17	0.09	0.03	0.01	0	0	0
Ca-containing regimens	0.01	0.02	0.04	0.07	0.12	0.18	0.17	0.15	0.14	0.10
Ge-containing regimens	0	0	0.01	0.02	0.07	0.19	0.23	0.20	0.18	0.09
PE-containing regimens	0.04	0.05	0.07	0.14	0.17	0.17	0.13	0.09	0.08	0.06
E-containing regimens	0.09	0.07	0.05	0.07	0.10	0.10	0.09	0.08	0.14	0.21

Supplementary Figure 2. Subgroup Bayesian network meta-analysis for pathologic complete response (pCR). (**A**) The league table of comparisons. Data are presented as odds radio (OR) and 95% confidence intervals (CI). An OR>1 favors the column-defining treatment, and OR <1 favors the row-defining treatment. (**B**) Heatmap of the rank probability of the regimens for pCR. Rank 1 represents the best treatment and rank 10 represents the worst. Rank probabilities sum to one, both within a rank over treatments and within a treatment over ranks.

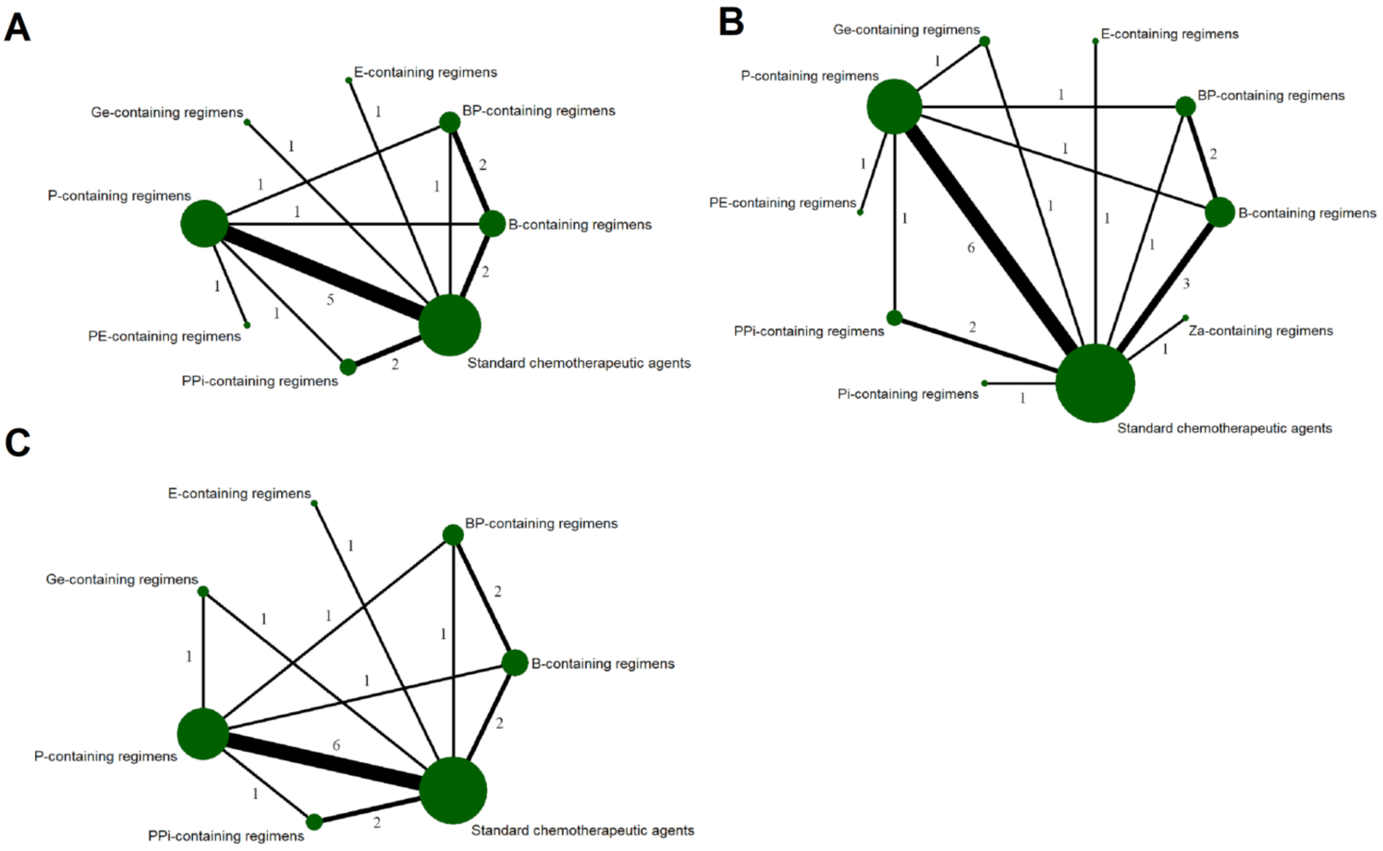

Supplementary Figure 3. Network diagram of eligible comparisons included in the network meta-analysis for serious adverse events. (**A**) Network diagram of eligible comparisons for anemia. (**B**) Network diagram of eligible comparisons for neutropenia. (**C**) Network diagram of eligible comparisons for thrombocytopenia. The node size is proportional to the total number of patients in the regimen. The width of each line is proportional to the number of studies comparing the two regimens linked by the line. B, bevacizumab; P, platinum salts; BP, bevacizumab plus platinum salts; Pi, Poly (ADP-ribose) polymerases inhibitors; PPi, platinum salts plus Poly (ADP-ribose) polymerases inhibitors; Ca, capecitabine; Ge, gemcitabine; Za, zoledronic acid; E, everolimus; PE, platinum salts plus everolimus; G, gefitinib.

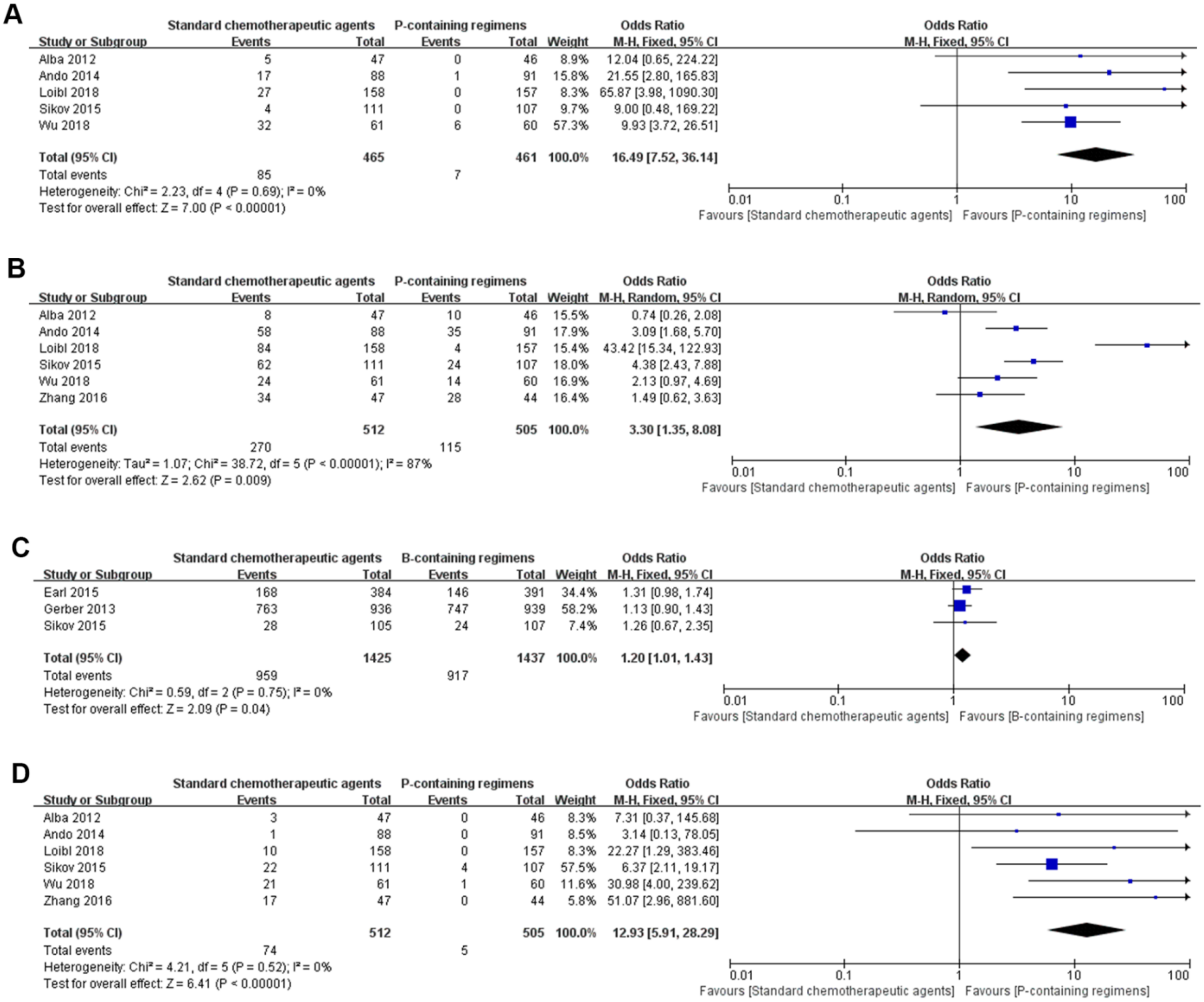

Supplementary Figure 4. Pairwise meta-analyses of serious adverse events. (**A**) Forest plot for anemia. Standard chemotherapeutic agents vs. P-containing regimens. (**B**) Forest plot for neutropenia. Standard chemotherapeutic agents vs. P-containing regimens. (**C**) Forest plot for neutropenia. Standard chemotherapeutic agents vs. B-containing regimens. (**D**) Forest plot for thrombocytopenia. Standard chemotherapeutic agents vs. P-containing regimens.

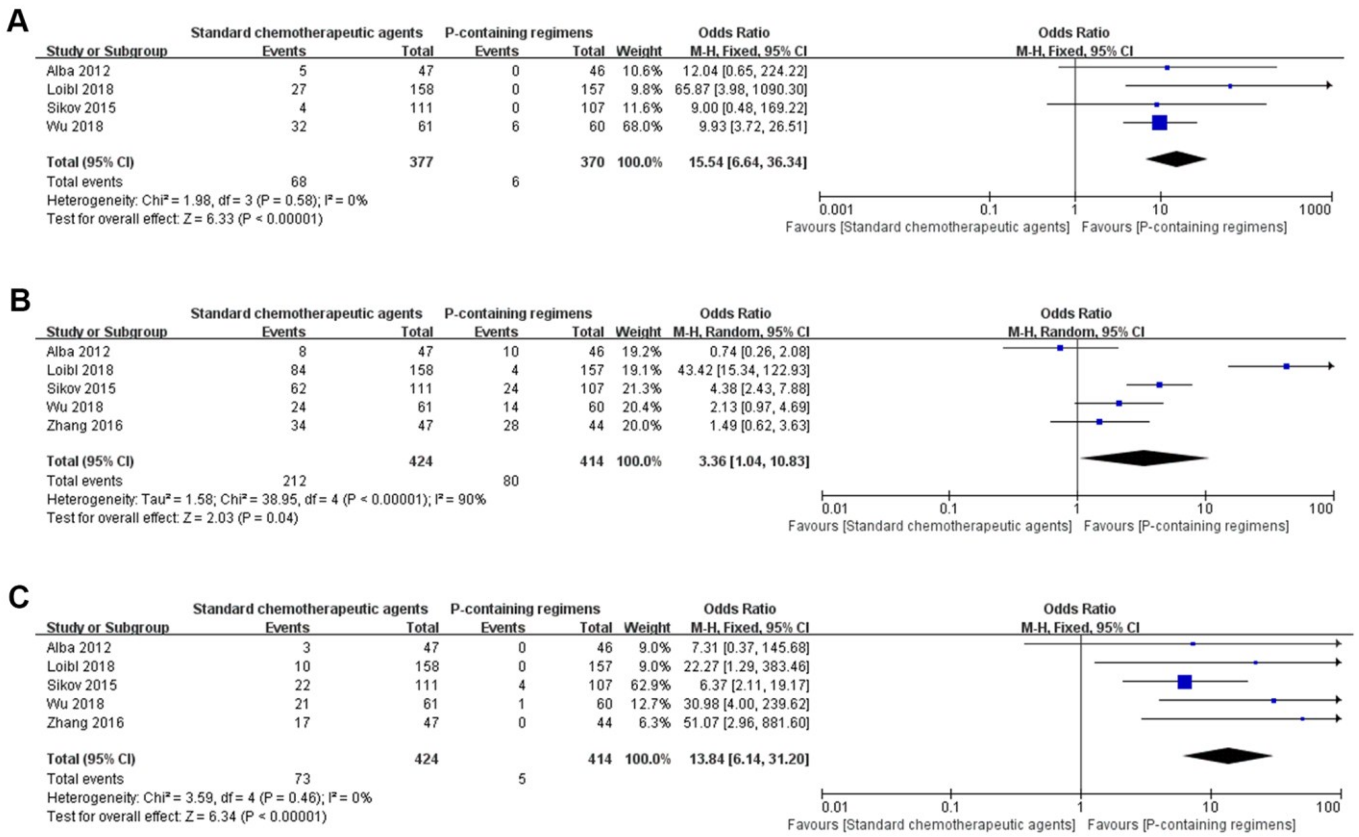

Supplementary Figure 5. Subgroup pairwise meta-analyses of serious adverse events in TNBC patients. (**A**) Forest plot for anemia. (**B**) Forest plot for neutropenia. (**C**) Forest plot for thrombocytopenia. The three comparisons are standard chemotherapeutic agents vs. P-containing regimens.

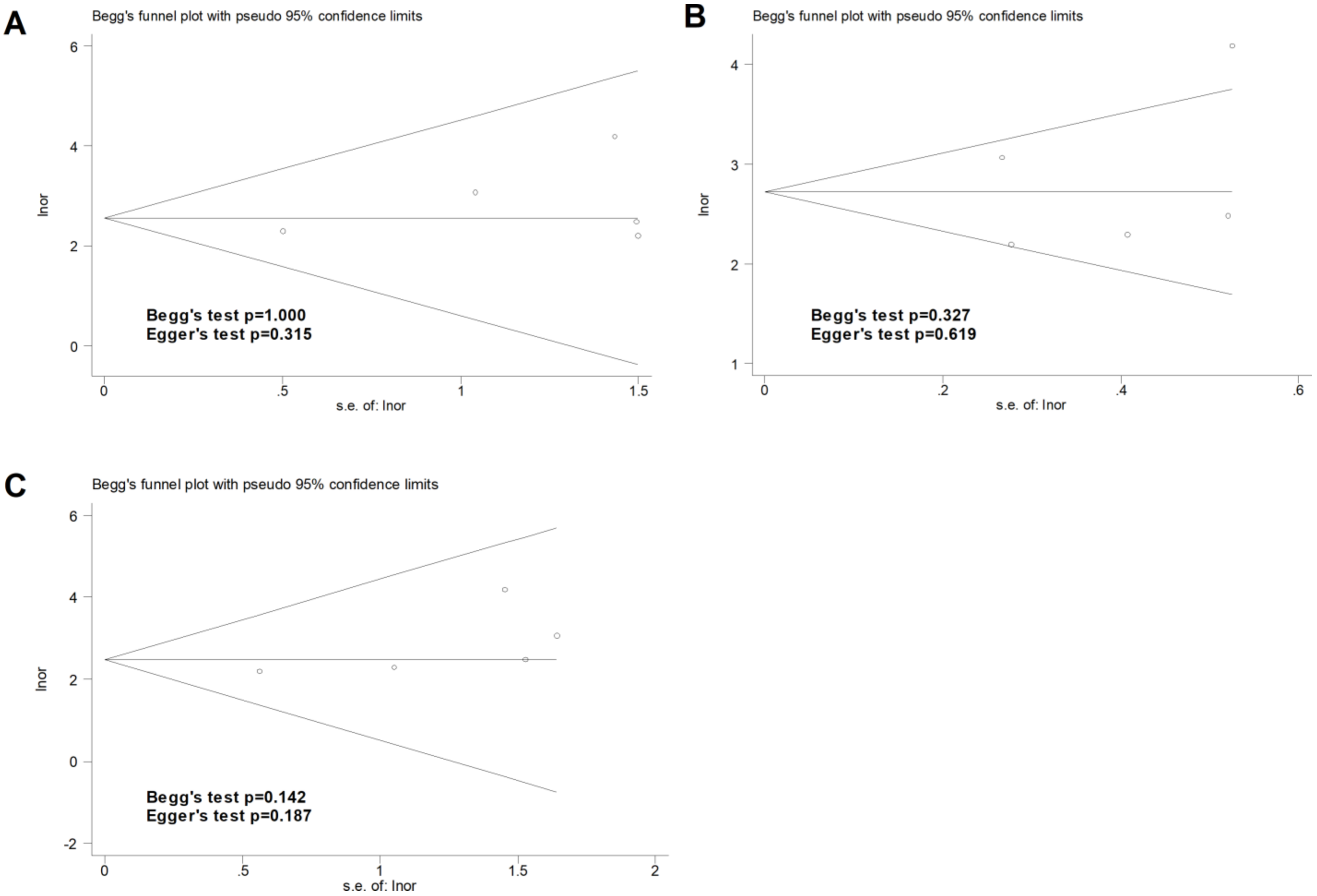

Supplementary Figure 6. Funnel plots of the publication bias tests for direct comparisons of serious adverse events. (**A**) Funnel plot for anemia. (**B**) Funnel plot for neutropenia. (**C**) Funnel plot for thrombocytopenia. The three comparisons are standard chemotherapeutic agents vs. P-containing regimens.

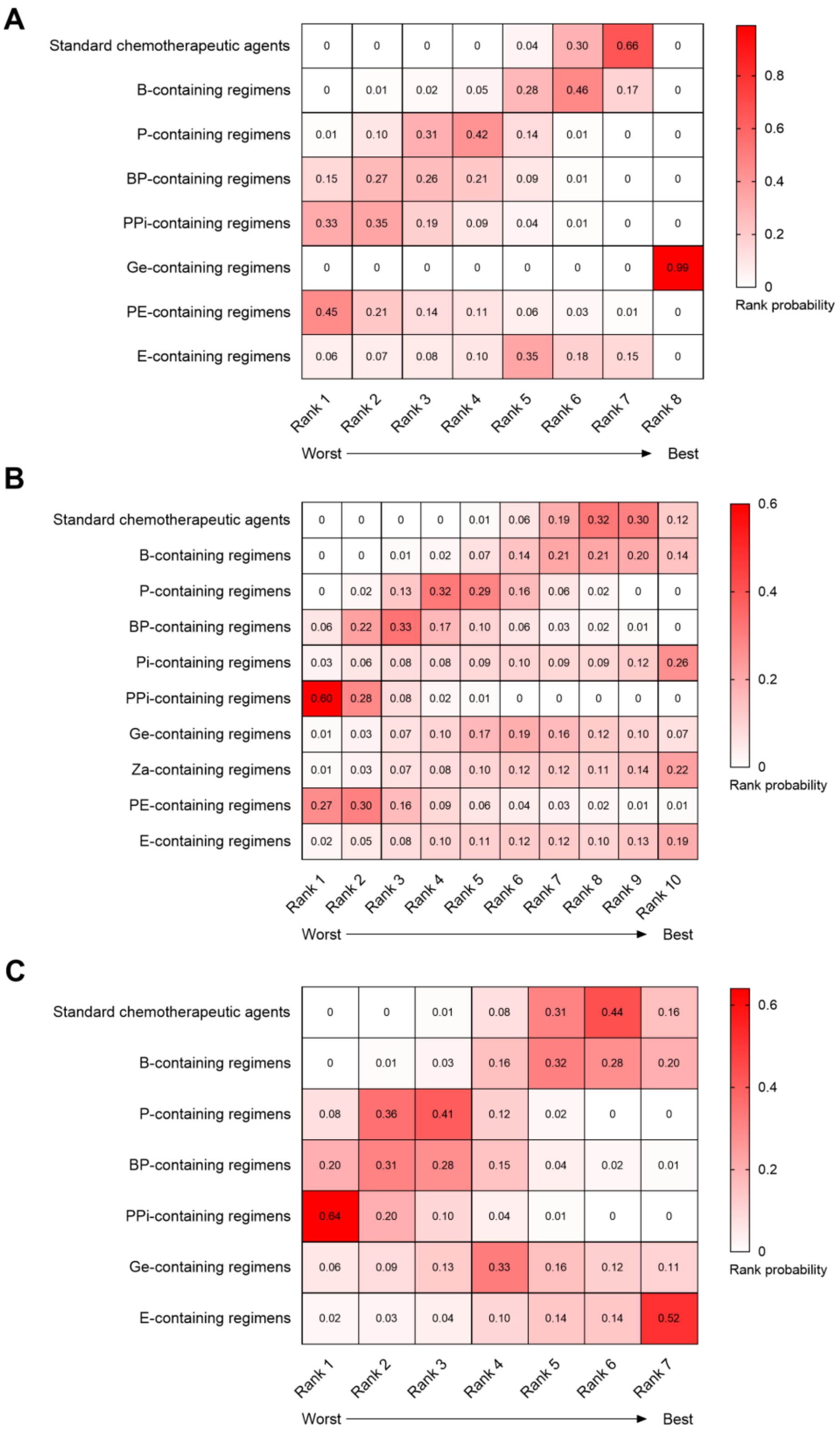

Supplementary Figure 7. Heatmaps of the rank probability of the regimens for serious adverse events. (**A**) Rank probability of anemia. (**B**) Rank probability of neutropenia. (**C**) Rank probability of thrombocytopenia. Rank 1 represents the worst treatment and rank *N* represents the best. Rank probabilities sum to one, both within a rank over treatments and within a treatment over ranks.

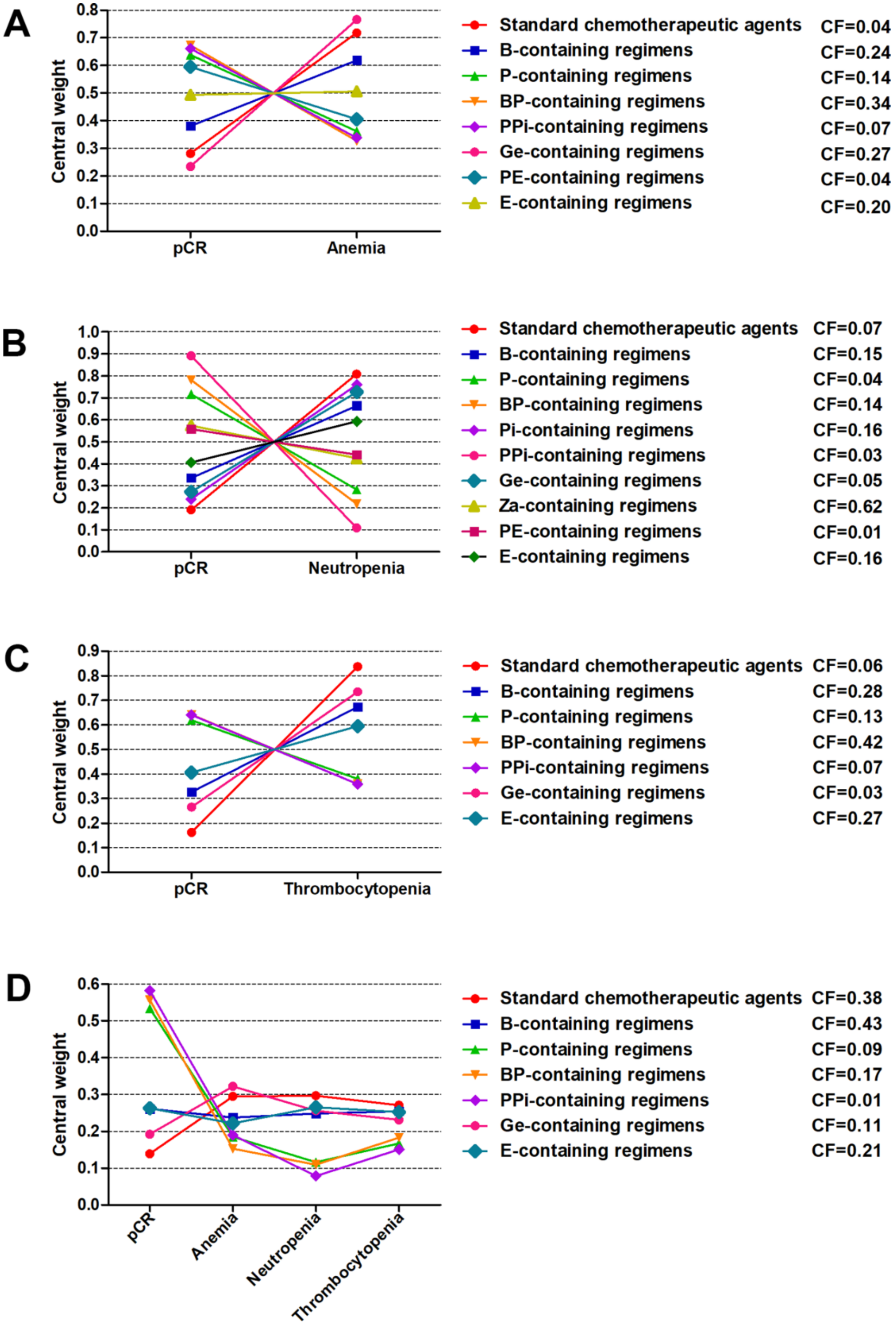

Supplementary Figure 8. Central weights and confidence factor (CF) for each regimen. (**A**) Analysis based on synthesizing pCR and anemia. (**B**) Analysis based on synthesizing pCR and neutropenia. (**C**) Analysis based on synthesizing pCR and thrombocytopenia. (**D**) Analysis based on synthesizing pCR and the three serious adverse events. The sum of the central weight for each intervention equals to one. A high central weight indicates that the intervention being considered is the best for that outcome. The confidence factor represents the probability of an intervention obtaining the first rank.
