# Supplementary material for: Efficacy and safety of neoadjuvant chemotherapy regimens for triple-negative breast cancer: a network meta-analysis
Source: Aging (Albany NY). 2019 Aug 24;11(16):6286–311. doi: 10.18632/aging.102188 (PMC6738404; doi:10.18632/aging.102188)
Supplement: Supplementary Table 1 [file aging-11-102188-s001.doc]

| **Supplementary Table 1. Treatment characteristics of the eligible studies.** | | | | | |
| --- | --- | --- | --- | --- | --- |
| Study | Arm design | NACT regimens | Agents administration dosage | Sample size in each arm | No. of pCR |
| Aft 2010 | Standard chemotherapetic agents vs. Za-containing regimens | TA | Docetaxel=75 mg/m^2^, Epirubicin=75 mg/m^2^ | 19 | 2* |
|  |  | TAZa | Docetaxel=75 mg/m^2^, Epirubicin =75 mg/m2, Zoledronic acid= 4mg | 21 | 6* |
| Houber 2010 | Standard chemotherapetic agents vs. Ca-containing regimens | TAC | Docetaxel=75 mg/m^2^, Epirubicin=50 mg/m^2^, Cyclophosphamide=500 mg/m^2^ | 48 | 6 |
|  |  | TACCa | Docetaxel=75 mg/m^2^, Epirubicin=90 mg/m^2^, Cyclophosphamide=600 mg/m^2^, Capecitabine=1000 mg/m^2^ | 41 | 2 |
| Bernsdorf 2011 | Standard chemotherapetic agents vs. G-containing regimens | AC | Epirubicin=90 mg/m^2^, Cyclophosphamide=600 mg/m^2^ | 41 | 5* |
|  |  | ACG | Epirubicin=90 mg/m^2^, Cyclophosphamide=600 mg/m^2^, Gefitinib=250 mg | 41 | 7* |
| Alba 2012 | Standard chemotherapetic agents vs. P-containing regimens | TAC | Docetaxel=100 mg/m^2^, Epirubicin=90 mg/m^2^, Cyclophosphamide=600 mg/m^2^ | 46 | 14 |
|  |  | TACP | Docetaxel=75 mg/m^2^, Epirubicin=90 mg/m^2^, Cyclophosphamide=600 mg/m^2^, Carboplatin=6 AUC | 47 | 14 |
| Gerber 2013 | Standard chemotherapetic agents vs. B-containing regimens | TAC | Docetaxel=100 mg/m2, Epirubicin=90 mg/m^2^, Cyclophosphamide=600 mg/m^2^ | 340 | 112 |
|  |  | TACB | Docetaxel=100 mg/m^2^, Epirubicin=90 mg/m^2^, Cyclophosphamide=600 mg/m^2^, Bevacizumab=15 mg/Kg | 323 | 140 |
| Steger 2014 | Standard chemotherapetic agents vs. Ca-containing regimens | TA | Docetaxel=75 mg/m^2^, Epirubicin=75 mg/m^2^ | 63 | 18 |
|  |  | TACa | Docetaxel=75 mg/m^2^, Epirubicin=75 mg/m^2^, Capecitabine=1000 mg/m^2^ | 64 | 26 |
| Ando 2014 | Standard chemotherapetic agents vs. P-containing regimens | TACF | Paclitaxel =80 mg/m^2^, Epirubicin=100 mg/m^2^, Cyclophosphamide=500 mg/m^2^, Florouracil=500 mg/m^2^ | 38 | 10 |
|  |  | TACFP | Paclitaxel=80 mg/m^2^, Epirubicin=100 mg/m^2^, Cyclophosphamide=500 mg/m^2^, Florouracil=500 mg/m^2^, Carboplatin=5 AUC | 37 | 23 |
| Earl 2014 | Standard chemotherapetic agents vs. Ge-containing regimens | TAC | Paclitaxel =175 mg/m^2^, Epirubicin=90 mg/m^2^, Cyclophosphamide=600 mg/m^2^ | 73 | 23 |
|  |  | TACGe | Paclitaxel =175mg/m^2^, Epirubicin=90mg/m^2^, Cyclophosphamide=600mg/m^2^, Gemcitabine=2000 mg/m^2^ | 84 | 27 |
| Gonzalez-Angulo 2014 | Standard chemotherapetic agents vs. E-containing regimens | TACF | Paclitaxel=80 mg/m^2^, Epirubicin=100mg/m^2^, Cyclophosphamide=500 mg/m^2^, Florouracil=500 mg/m^2^ | 27 | 7 |
|  |  | TACFE | Paclitaxel=80 mg/m^2^, Epirubicin=100 mg/m^2^, Cyclophosphamide=500 mg/m^2^, Florouracil=500 mg/m^2^, Everolimus=30 mg | 23 | 7 |
| von Minckwitz 2014 | B-containing regimens vs. BP-containing regimens | TAB | Paclitaxel=80 mg/m^2^, Doxorubicin=20 mg/m^2^, Bevacizumab=15 mg/kg | 157 | 67 |
|  |  | TABP | Paclitaxel =80 mg/m^2^, Doxorubicin=20mg/m^2^, Bevacizumab=15 mg/kg, Carboplatin=2 AUC | 158 | 90 |
| Earl 2015 | Standard chemotherapetic agents vs. B-containing regimens | TACF | Docetaxel=100 mg/m^2^, Epirubicin=100 mg/m^2^, Cyclophosphamide=500 mg/m^2^, Florouracil=500 mg/m^2^ | 122 | 38 |
|  |  | TACFB | Docetaxel=100 mg/m^2^, Epirubicin=100 mg/m^2^, Cyclophosphamide=500 mg/m^2^, Florouracil=500 mg/m^2^, Bevacizumab=15mg/kg | 119 | 54 |
| Hasegawa 2015 | Standard chemotherapetic agents vs. Za-containing regimens | TACF | Paclitaxel=80 mg/m^2^, Epirubicin=100 mg/m^2^, Cyclophosphamide=500 mg/m^2^, Florouracil=500 mg/m^2^ | 17 | 2* |
|  |  | TACFZa | Paclitaxel=80mg/m^2^, Epirubicin=100mg/m^2^, Cyclophosphamide=500mg/m^2^, Florouracil=500mg/m^2^, Zoledronic acid=4 mg | 17 | 6* |
| Llombart-Cussac 2015 | Standard chemotherapetic agents vs. Pi-containing regimens | T | Paclitaxel=80 mg/m^2^ | 46 | 10 |
|  |  | TPi | Paclitaxel=80 mg/m^2^, Iniparib=5.6/12.2 mg/kg | 94 | 17 |
| Martinez 2015 | Standard chemotherapetic agents vs. P-containing regimens | TACF | Paclitaxel=80 mg/m^2^, Doxorubicin=50 mg/m^2^, Cyclophosphamide=500 mg/m^2^, Florouracil=500 mg/m^2^ | 31 | 12 |
|  |  | TAP | Paclitaxel=80 mg/m^2^, Doxorubicin=50 mg/m^2^, Cisplatin=30 mg/m^2^ | 30 | 18 |
| Sikov 2015 | Standard chemotherapetic agents vs. B-containing regimens vs. P-containing regimens vs. BP-containing regimens | TAC | Paclitaxel=80 mg/m^2^, Doxorubicin=60 mg/m^2^, Cyclophosphamide=600 mg/m^2^ | 107 | 42 |
|  |  | TACB | Paclitaxel=80 mg/m^2^, Doxorubicin=60 mg/m^2^, Cyclophosphamide=500 mg/m^2^, Bevacizumab=10 mg/kg | 105 | 45 |
|  |  | TACP | Paclitaxel=80mg/m^2^, Doxorubicin=60 mg/m^2^, Cyclophosphamide= 500mg/m^2^, Carboplatin=6 AUC | 111 | 54 |
|  |  | TACBP | Paclitaxel=80 mg/m^2^, Doxorubicin=60 mg/m^2^, Cyclophosphamide= 500mg/m^2^, Bevacizumab=10 mg/kg, Carboplatin=6 AUC | 110 | 66 |
| Nahleh 2016 | Standard chemotherapetic agents vs. B-containing regimens | TAC | nab-Paclitaxel =100 mg/m^2^, Doxorubicin=60 mg/m^2^, Cyclophosphamide=600 mg/m^2^ | 35 | 10 |
|  |  | TACB | nab-Paclitaxel=100 mg/m^2^, Doxorubicin=60 mg/m^2^, Cyclophosphamide=600 mg/m^2^, Bevacizumab=10 mg/kg | 32 | 19 |
| Zhang 2016 | Standard chemotherapetic agents vs. P-containing regimens | TA | Paclitaxel=175 mg/m^2^, Epirubicin=75 mg/m^2^ | 43 | 6 |
|  |  | TP | Paclitaxel=175 mg/m^2^, Carboplatin=5 AUC | 44 | 17 |
| Rugo 2016 | Standard chemotherapetic agents vs. PPi-containing regimens | TAC | Paclitaxel=80mg/m2, Doxorubicin=60mg/m^2^, Cyclophosphamide=600mg/m2 | 21 | 5 |
|  |  | TACPPi | Paclitaxel=80 mg/m^2^, Doxorubicin=60 mg/m^2^, Cyclophosphamide=600 mg/m^2^, Carboplatin=6 AUC, Veliparib=50 mg/m^2^ | 39 | 20 |
| Enriquez 2017 | Standard chemotherapetic agents vs. P-containing regimens | TAC | Paclitaxel=80 mg/m^2^, Adriamycin=60 mg/m^2^, Cyclophosphamide=600 mg/m^2^ | 34 | 8 |
|  |  | TP | Docetaxel=75mg/m^2^, Carboplatin=6 AUC | 27 | 10 |
| Gluz 2017 | Ge-containing regimens vs. P-containing regimens | TGe | nab-Paclitaxel =125 mg/m^2^, Gemcitabine=1000 mg/m^2^ | 178 | 51 |
|  |  | TP | nab-Paclitaxel =125 mg/m^2^, Carboplatin=2 AUC | 146 | 67 |
| Jovanović 2017 | P-containing regimens vs. PE-containing regimens | TP | Paclitaxel=80 mg/m^2^, Cisplatin=25 mg/m^2^ | 49 | 24 |
|  |  | TPE | Paclitaxel=80 mg/m^2^, Cisplatin=25 mg/m^2^, Everolimus= 30mg | 96 | 35 |
| Loibl 2018 | Standard chemotherapetic agents vs. P-containing regimens vs. PPi-containing regimens | TAC | Paclitaxel=80 mg/m^2^, Doxorubicin=60 mg/m^2^, Cyclophosphamide=600 mg/m^2^ | 158 | 49 |
|  |  | TACP | Paclitaxel=80 mg/m^2^, Doxorubicin=60 mg/m^2^, Cyclophosphamide=600 mg/m^2^, Carboplatin=6 AUC | 160 | 92 |
|  |  | TACPPi | Paclitaxel=80 mg/m^2^, Doxorubicin=60 mg/m^2^, Cyclophosphamide=600 mg/m^2^, Carboplatin=6 AUC, Veliparib=50 mg/m^2^ | 316 | 168 |
| Wu 2018 | Standard chemotherapetic agents vs. P-containing regimens | TA | Docetaxel=75 mg/m^2^, Epirubicin=80 mg/m^2^ | 60 | 8 |
|  |  | TAP | Docetaxel=75mg/m^2^, Epirubicin=80mg/m^2^, Lobaplatin=30 mg/m^2^ | 61 | 24 |
| A, anthracyclines; B, bevacizumab; C, cyclophosphamide; Ca, capecitabine; E, everolimus; F, florouracil; G, gefitinib; Ge, gemcitabine; P, platinum salts; Pi,Poly(ADP-ribose) polymerases (PARPs); T, taxanes; Za, zoledronic acid; NA, not available; *pCR defined as ypT0/is. | | | | | |
