# Supplementary material for: Efficacy and safety of neoadjuvant chemotherapy regimens for triple-negative breast cancer: a network meta-analysis
Source: Aging (Albany NY). 2019 Aug 24;11(16):6286–311. doi: 10.18632/aging.102188 (PMC6738404; doi:10.18632/aging.102188)
Supplement: Supplementary Tables 2 and 3 [file aging-11-102188-s002.pdf]

SUPPLEMENTARY TABLES

Please browse Full Text version to see the data of Supplementary Table 1

Supplementary Table 1. Treatment characteristics of the eligible studies.

Supplementary Table 2. Risk of bias assessments for included trials.

Study	Selection bias-Random sequence generation	Selection bias-Allocation concealment	Performance bias-Blinding of participants and personnel	Detection bias-Blinding of outcome assessment	Attrition bias-Incomplete outcome data	Reporting bias-Selective reporting	Other bias
Aft 2010	+	+	+	+	+	+	?
Houber 2010	?	?	+	+	+	+	+
Bernsdorf 2011	?	?	+	+	+	+	?
Alba 2012	+	+	+	+	+	+	+
Gerber 2013	?	?	+	+	+	+	+
Ando 2014	?	?	+	+	+	+	+
Earl 2014	+	+	+	+	+	+	+
Gonzalez-Angulo 2014	+	+	+	+	+	+	+
Steger 2014	+	?	+	+	+	+	+
von Minckwitz 2014	+	+	+	+	+	+	+
Earl 2015	+	+	+	+	+	+	+
Hasegawa 2015	+	+	+	+	+	+	?
Llombart-Cussac 2015	?	?	+	+	+	+	+
Martinez 2015	?	?	+	+	+	?	+
Sikov 2015	?	?	+	+	+	+	+
Nahleh 2016	?	?	+	+	+	+	+
Zhang 2016	?	?	+	+	+	+	+
Rugo 2016	+	+	+	+	+	+	+
Enriquez 2017	-	-	+	+	+	+	+
Gluz 2017	?	+	+	+	+	+	+
Jovanović 2017	+	+	+	+	+	+	+
Loibl 2018	+	+	+	+	+	+	+
Wu 2018	+	?	+	+	+	+	+

+=low risk of bias; -=high risk of bias; ?=unclear risk of bias. Other bias is defined as the definition of pCR (i.e., whether it is defined clearly).

Supplementary Table 3. Grade 3-4 hematological adverse events.

Study	Arm design	NACT regimens	BC patients	No. of patients	Anemia	Neutropenia	Thrombocytopenia
Aft 2010	Standard chemotherapeutic agents vs. Za-containing regimens	TA	NA	NA	NA	NA	NA
		TAZa	NA	NA	NA	NA	NA
Houber 2010	Standard chemotherapeutic agents vs. Ca-containing regimens	TAC	NA	NA	NA	NA	NA
		TACCa	NA	NA	NA	NA	NA
Bernsdorf 2011	Standard chemotherapeutic agents vs. G-containing regimens	AC	ER-	NA	NA	NA	NA
		ACG	ER-	NA	NA	NA	NA
Alba 2012	Standard chemotherapeutic agents vs. P-containing regimens	TAC	TNBC	46	0	10	0
		TACP	TNBC	47	5	8	3
Gerber 2013	Standard chemotherapeutic agents vs. B-containing regimens	TAC	Her2-	963	16/963	747/939	15/963
		TACB	Her2-	951	17/950	763/936	20/951
Steger 2014	Standard chemotherapeutic agents vs. Ca-containing regimens	TA	NA	NA	NA	NA	NA
		TACa	NA	NA	NA	NA	NA
Ando 2014	Standard chemotherapeutic agents vs. P-containing regimens	TACF	Her2-	91	1	35	0
		TACFP	Her2-	88	17	58	1
Earl 2014	Standard chemotherapeutic agents vs. Ge-containing regimens	TAC	All	404	3	135	2
		TACGe	All	408	0	146	0
Gonzalez-Angulo 2014	Standard chemotherapeutic agents vs. E-containing regimens	TACF	TNBC	27	1	11	2
		TACFE	TNBC	23	4	12	1
von Minckwitz 2014	B-containing regimens vs. BP-containing regimens	TAB	TNBC/Her2+	293	1	79	1
		TABP	TNBC/Her2+	295	45	192	42
Earl 2015	Standard chemotherapeutic agents vs. B-containing regimens	TACF	All	391	NA	146	NA
		TACFB	All	384	NA	168	NA
Hasegawa 2015	Standard chemotherapeutic agents vs. Za-containing regimens	TACF	Her2-	95	NA	34	NA
		TACFZa	Her2-	91	NA	39	NA
Llombart-Cussac 2015	Standard chemotherapeutic agents vs. Pi-containing regimens	T	TNBC	46	NA	2	NA
		TPi	TNBC	94	NA	5	NA
Martinez 2015	Standard chemotherapeutic agents vs. P-containing regimens	TACF	NA	NA	NA	NA	NA
		TAP	NA	NA	NA	NA	NA
Sikov 2015	Standard chemotherapeutic agents vs. B-containing regimens vs. P-containing regimens vs. BP-containing	TAC	TNBC	107	0	24	4
		TACB	TNBC	105	2	28	3
		TACP	TNBC	111	4	62	22
		TACBP	TNBC	110	6	74	29

	regimens						
Nahleh 2016	Standard chemotherapeutic agents vs. B-containing regimens	TAC	NA	NA	NA	NA	NA
		TACB	NA	NA	NA	NA	NA
Zhang 2016	Standard chemotherapeutic agents vs. P-containing regimens	TA	TNBC	44	NA	28	0
		TP	TNBC	47	NA	34	17
Rugo 2016	Standard chemotherapeutic agents vs. PPi-containing regimens	TAC	Her2-	44	0	1	0
		TACPPi	Her2-	72	20	51	15
Enriquez 2017	Standard chemotherapeutic agents vs. P-containing regimens	TAC	NA	NA	NA	NA	NA
		TP	NA	NA	NA	NA	NA
Gluz 2017	Ge-containing regimens vs. P-containing regimens	TGe	TNBC	180	NA	29	2
		TP	TNBC	151	NA	24	0
Jovanović 2017	P-containing regimens vs. PE-containing regimens	TP	TNBC	49	1	5	0
		TPE	TNBC	96	6	25	0
Loibl 2018	Standard chemotherapeutic agents vs. P-containing regimens vs. PPi-containing regimens	TAC	TNBC	157	0	4	0
		TACP	TNBC	158	27	84	10
		TACPPi	TNBC	313	77	179	33
Wu 2018	Standard chemotherapeutic agents vs. P-containing regimens	TA	TNBC	60	6	14	1
		TAP	TNBC	61	32	24	21

A, anthracyclines; B, bevacizumab; C, cyclophosphamide; Ca, capecitabine; E, everolimus; F, florouracil; G, gefitinib; Ge, gemcitabine; P, platinum salts; Pi,Poly(ADP-ribose) polymerases (PARPs); T, taxanes; Za, zoledronic acid; NA, not available.
